# Supplementary figures and images for: Reduced folate carrier 1 is present in retinal microvessels and crucial for the inner blood retinal barrier integrity
Source: Fluids Barriers CNS. 2023 Jun 16;20:47. doi: 10.1186/s12987-023-00442-3 (PMC10276430; doi:10.1186/s12987-023-00442-3)

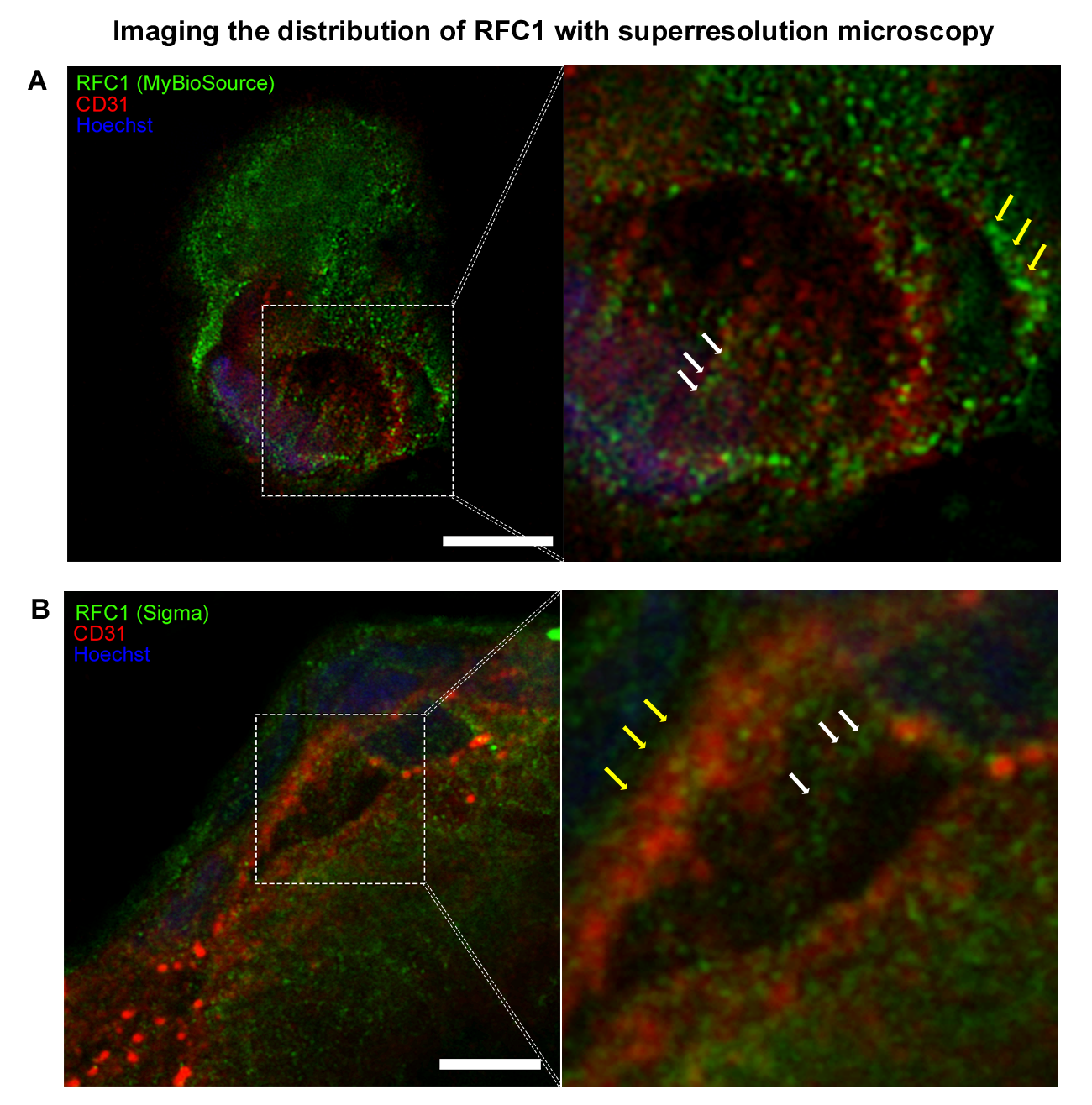

Supplement: Supplementary file 1 — Additional file 1: Fig. S1. Super-resolution microscopy of retinal radial sections to reveal the distribution of RFC1 protein over the endothelial cell surface. (A) 3D reconstruction of RFC1 immunostaining performed by MyBioSource antibody which is targeted to N-terminal region of protein. (B) 3D reconstruction of RFC1 immunostaining performed by Sigma antibody which is targeted to C-terminal region of the protein. In both stainings, RFC1 puncta are localized to the luminal endothelial cell surface stained by CD31 (white arrows). RFC1 puncta are also visible from the abluminal side (yellow arrows), indicating abluminal endothelial membrane or pericyte localization of RFC1 protein. Scale bars=10 µm. [file 12987_2023_442_MOESM1_ESM.tif]

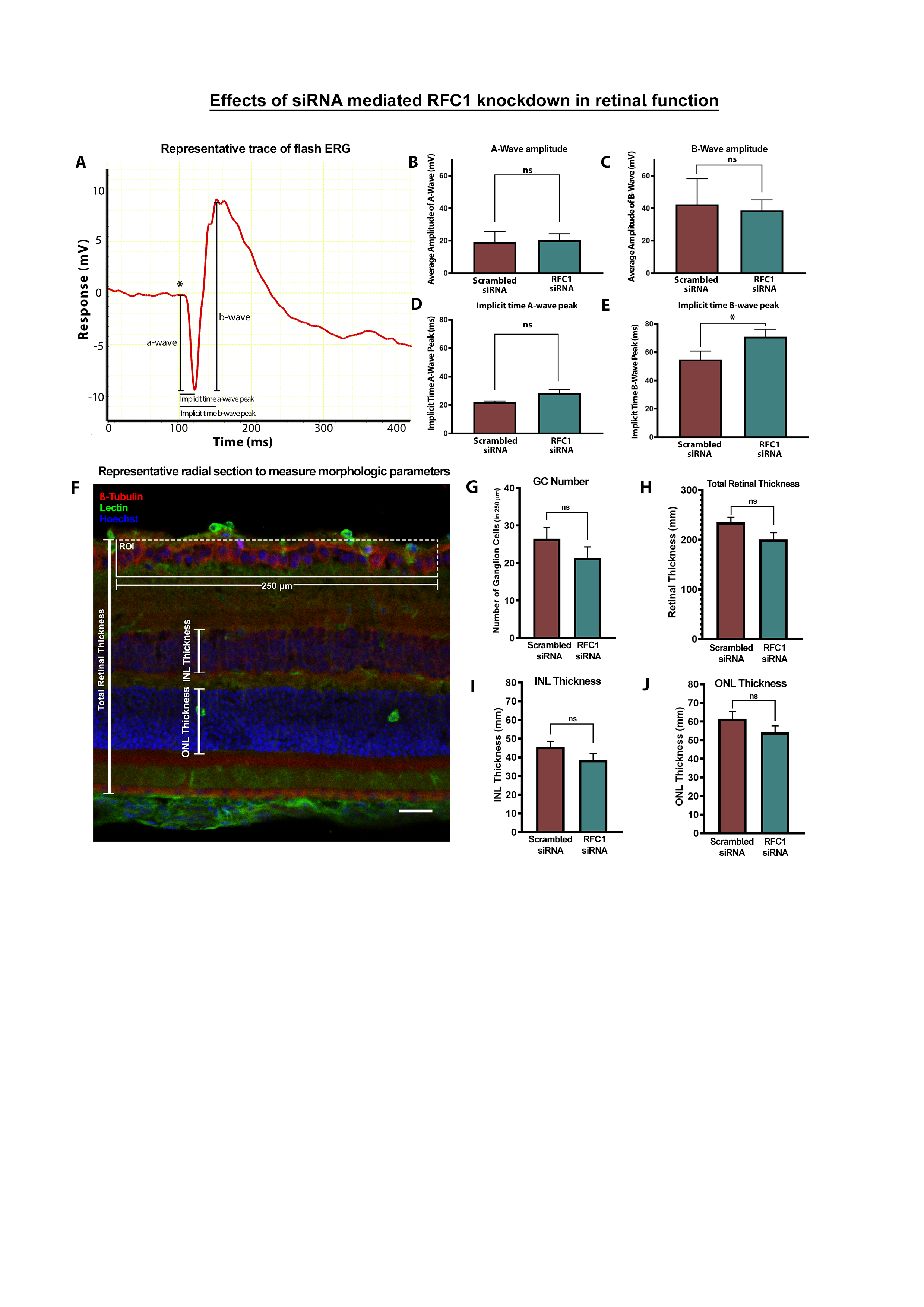

Supplement: Supplementary file 2 — Additional file 2: Fig S2. Effects of siRNA mediated RFC1 knockdown in the retina. A Representative scotopic (dark-adapted) electroretinogram (ERG). The ERG recordings include measurements of the a-wave, b-wave, and implicit time of a-wave peak and b-wave peak. The amplitude of the a-wave is determined by measuring the distance between the baseline and the lowest point of the negative deflection. On the other hand, the amplitude of the b-wave is calculated by measuring the distance between the maximum point of the a-wave and the peak of the positive deflection of the b-wave. 0 mV indicates baseline response. B, C Comparison of mean amplitudes ± SEM of scotopic a-wave and b-wave between Scrambled-siRNA and RFC1-siRNA treated mice. D, E Comparison of mean implicit time ± SEM of A-wave peak and B-wave peak between scrambled-siRNA and RFC1-siRNA mice. Only average of the implicit time B-wave peak between Scrambled-siRNA (55±3.0 ms) and RFC1-siRNA (71±2.7 ms) was different (p=0.029; n=4/group). F β-Tubulin III (red), Lectin (green), and Hoechst 33258 (blue) stained central retinal cross-sections were imaged with confocal microscopy. To manually count GC number, ROIs in 250 µm length were placed on the ganglion cell layer. Total retinal thickness was determined from the nerve fiber layer to retinal pigment epithelium. The width of the INL (the distance between the inner plexiform layer and the outer plexiform layer) and ONL (the distance between the outer plexiform layer and the outer limiting membrane) were determined by a fluorescent nuclear dye (Hoechst 33258). G-J The bar graphs show that there is no statistically significant difference in the abovementioned parameters between RFC1-siRNA and Scrambled-siRNA treated groups (n=3/per group). *p=0.029, data are mean ± S.E.M Mann-Whitney U; Scale bar: 25 μm. GC: ganglion cell, INL: inner nuclear layer, ONL: outer nuclear layer, ROI: Region of interest. [file 12987_2023_442_MOESM2_ESM.tif]
